# Updating high-resolution image dataset for the automatic classification of phenological stage and identification of racemes in *Urochloa* spp. hybrids with expanded images and annotations

**DOI:** 10.1016/j.dib.2025.111593

**Published:** 2025-04-28

**Authors:** Darwin Alexis Arrechea-Castillo, Paula Espitia-Buitrago, David Florian-Vargas, Ronald David Arboleda, Riquelmer Velázquez-Hernández, Andrés Felipe Ruiz-Hurtado, Luis Miguel Hernandez, Rosa N. Jauregui, Juan Andrés Cardoso

**Affiliations:** a International Center for Tropical Agriculture (CIAT), A.A. 6713, Km 17 recta Cali-Palmira, Palmira, Colombia; b Grupo Papalotla, C.P, Ocozocoautla – Cintalapa km 110, Col. El Aguacero, Ocozocoautla de Espinosa, Chiapas 29140, México

**Keywords:** Brachiaria, Forage grasses, Machine learning, Deep learning, Instance segmentation, Artificial intelligence, Computer vision, High-throughput phenotyping

## Abstract

This dataset is an expanded version of a previously published collection of high-resolution RGB images of *Urochloa spp.* genotypes, initially designed to facilitate automated classification of phenological stages and raceme identification in forage breeding trials. The original dataset included 2400 images of 200 genotypes captured under controlled conditions, supporting the development of computer vision models for High-Throughput Phenotyping (HTP). In this updated release, 139 additional images and 24,983 new annotations have been added, bringing the dataset to a total of 2539 images and 47,323 raceme annotations. This version introduces increased diversity in image-capture conditions, with data collected from two geographic locations (Palmira, Colombia, and Ocozocoautla de Espinosa, Mexico) and a range of image-capture devices, including smartphones (e.g. Realme C53 and Oppo Reno 11), a Nikon D5600 camera, and a Phantom 4 Pro V2 drone. Images now vary in perspective (nadir, high-angle, and frontal) and capture distance (1–3 meters), enhancing the dataset applicability for robust Deep Learning (DL) models. Compared to the original dataset, raceme density per plant has nearly doubled in some samples, offering higher raceme overlap for advanced instance segmentation tasks. This expanded dataset supports deeper exploration of phenotypic variation in *Urochloa* spp. and offers greater potential for developing adaptable models in crop phenotyping.

Specifications Table

**Table:** 

Subject	Agricultural Sciences. Computer Science.
Specific subject area	Agronomy and crop science. Artificial intelligence, computer science applications, computer vision and pattern recognition.
Type of data	Image Raw data in .JPG format. Racemes annotations are stored using s .json file in COCO “Common Objects in Context” format.
Data collection	Images were taken outdoors at different times of the day, resulting in varied lighting conditions. Photos were captured at two locations: Palmira, Colombia, and Ocozocoautla de Espinosa, Mexico. Six devices were used: a Nikon D5600 professional camera, Realme C53, Moto G7 Power, Galaxy A71 and Oppo Reno 11 smartphones, and a Phantom 4 Pro V2 drone. Capture perspectives varied (nadir, high-angle, and frontal), as did distances (1–3m). Some plants were captured in natural conditions with weeds (i.e., *Cynodon dactylon* and *Paspalum notatum*), providing complex backgrounds.
Data source location	Institution 1: Alliance Bioversity International & CIAT. Institution 2: Grupo Papalotla City 1: Palmira, Valle del Cauca. City 2: Ocozocoautla de Espinosa, Chiapas. Country 1: Colombia. Country 2: Mexico. Geolocalization 1: 3°29’N, 76°21’W Geolocalization 2: 16°45′N 93°28′W
Data accessibility	Repository name: Harvard Dataverse Data identification number: doi.org/10.7910/DVN/X4LM19 Direct URL to data: https://doi.org/10.7910/DVN/X4LM19 Instructions for accessing these data: The original dataset [1] and the updated dataset version [2], are both licensed under the Creative Commons Attribution 4.0 International, which allows use, sharing, adapting, distribution, and reproduction in any medium or format if attribution is given to the creator. The datasets can be downloaded without providing any personal information. The fields for “Name”, “Email”, “Institution”, “Position”, and “Intended use of the data” are entirely optional. By simply clicking the “Accept” button without filling in any fields, users can immediately access the data.
Related data article	Explore all the details in this Ref. [3]*.*

## 1 Value of the Data

• The expanded dataset introduces increased variability in image-capture conditions, including geographic locations, lighting, capture devices, and perspectives. Additionally, this dataset includes genotypes with different morphological characteristics that reflects the diversity of *Urochloa* spp. This diversity enhances the dataset robustness, allowing for the training of Machine Learning (ML) and Deep Learning (DL) models that are better adapted to real-world variations. Furthermore, the increased raceme density and complex backgrounds improve the dataset utility for developing advanced phenotyping algorithms, particularly for tasks requiring raceme instance segmentation and trait classification.
• Researchers in agriculture, computer vision, and plant phenotyping can benefit from this dataset. The variability in environmental and image-capture conditions makes it an ideal resource for developing and testing Artificial Intelligence (AI) models that require generalized data. Breeders and agronomists focusing on *Urochloa* spp. can also leverage on the dataset for High-Throughput Phenotyping (HTP), helping to accelerate breeding programs targeting complex traits such as forage seed yield.

## 2 Background

**Fig. 1:**
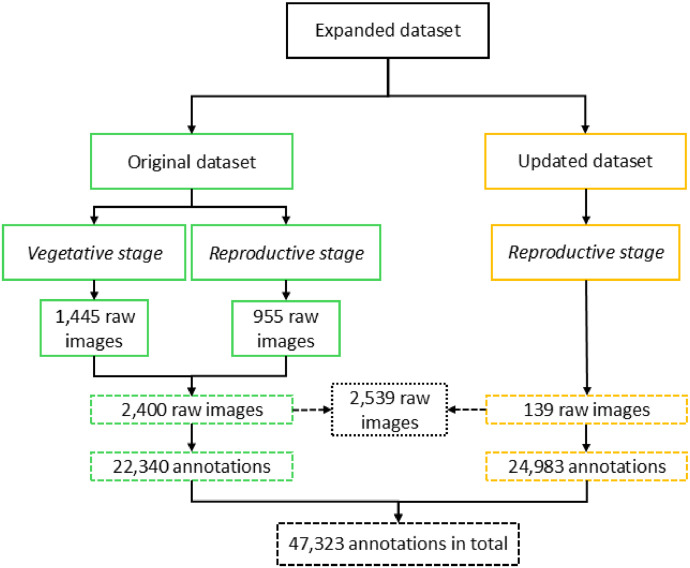
Comparison between the original dataset and the updated one.

Tropical forages, particularly *Urochloa* grasses, are essential for sustainable livestock production in the Neotropics and are gaining importance in Sub-Saharan Africa and South-East Asia [4]. These grasses are important to support production and play a crucial role in sustainable agriculture by thriving under diverse environmental conditions. One of the traits that *Urochloa* breeding program at CIAT aims to improve is seed yield, which requires HTP to accelerate breeding cycles and to increase genetic gain in the traits [3]. To achieve this, high-quality and diverse image datasets are essential, as they enable the development of DL models that can handle and adapt to varying field conditions [5]. The original dataset [1] contributed to this goal by providing researchers with 2,400 images and 22,340 raceme annotations, facilitating the initial development of ML and DL models for phenological stage classification and raceme instance segmentation in *Urochloa* spp. However, a need was identified to increase the diversity in imaging conditions including additional annotations to enhance model robustness and reflect real-world variation. To address this, an expanded dataset with 139 images and 24,983 new annotations was built (see Fig. 1). Providing a more comprehensive resource for developing robust computer vision models that support the breeding of *Urochloa spp*.

## 3 Data Description

**Fig. 2:**
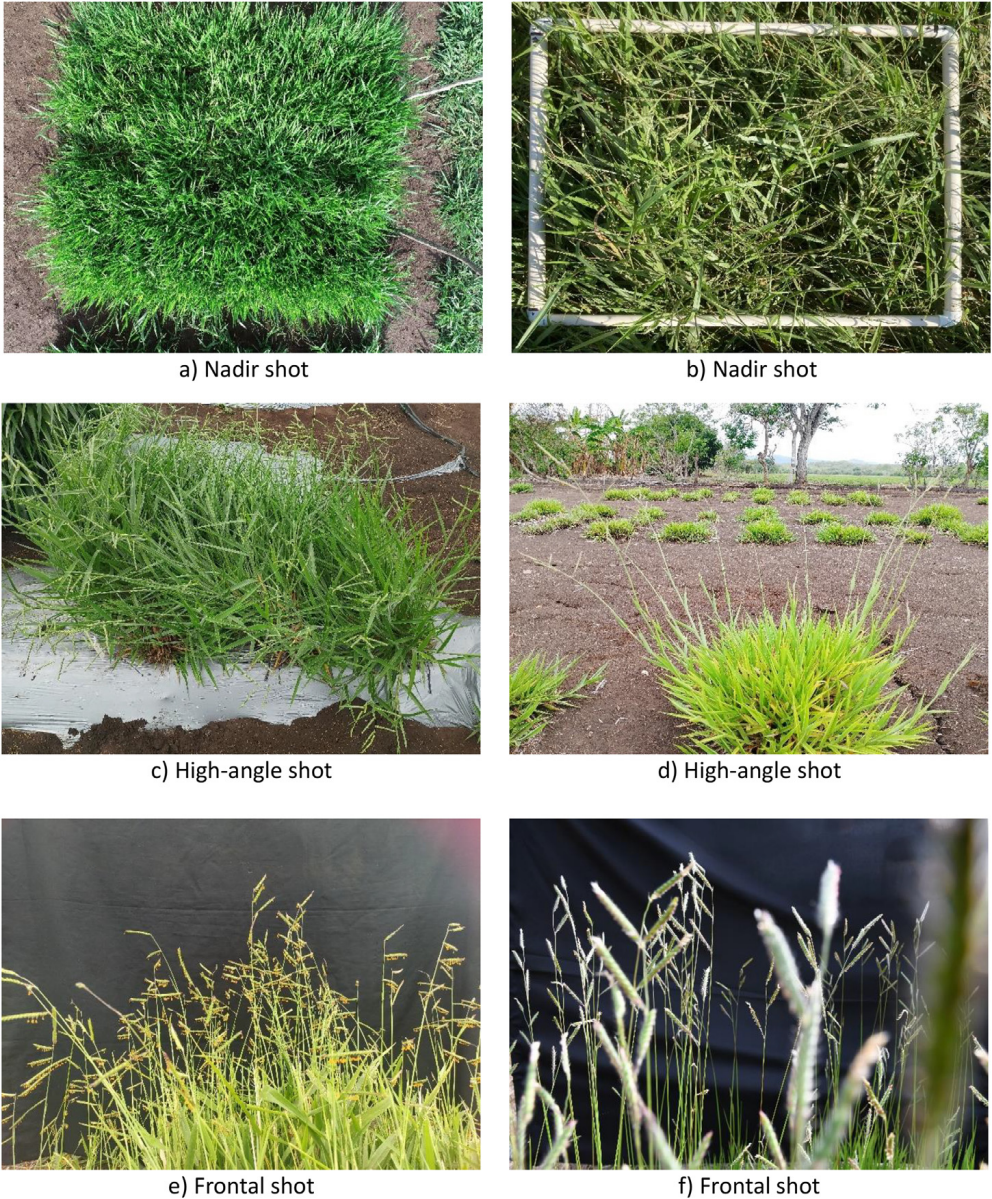
Subset of the raw captured images.

**Fig. 3:**
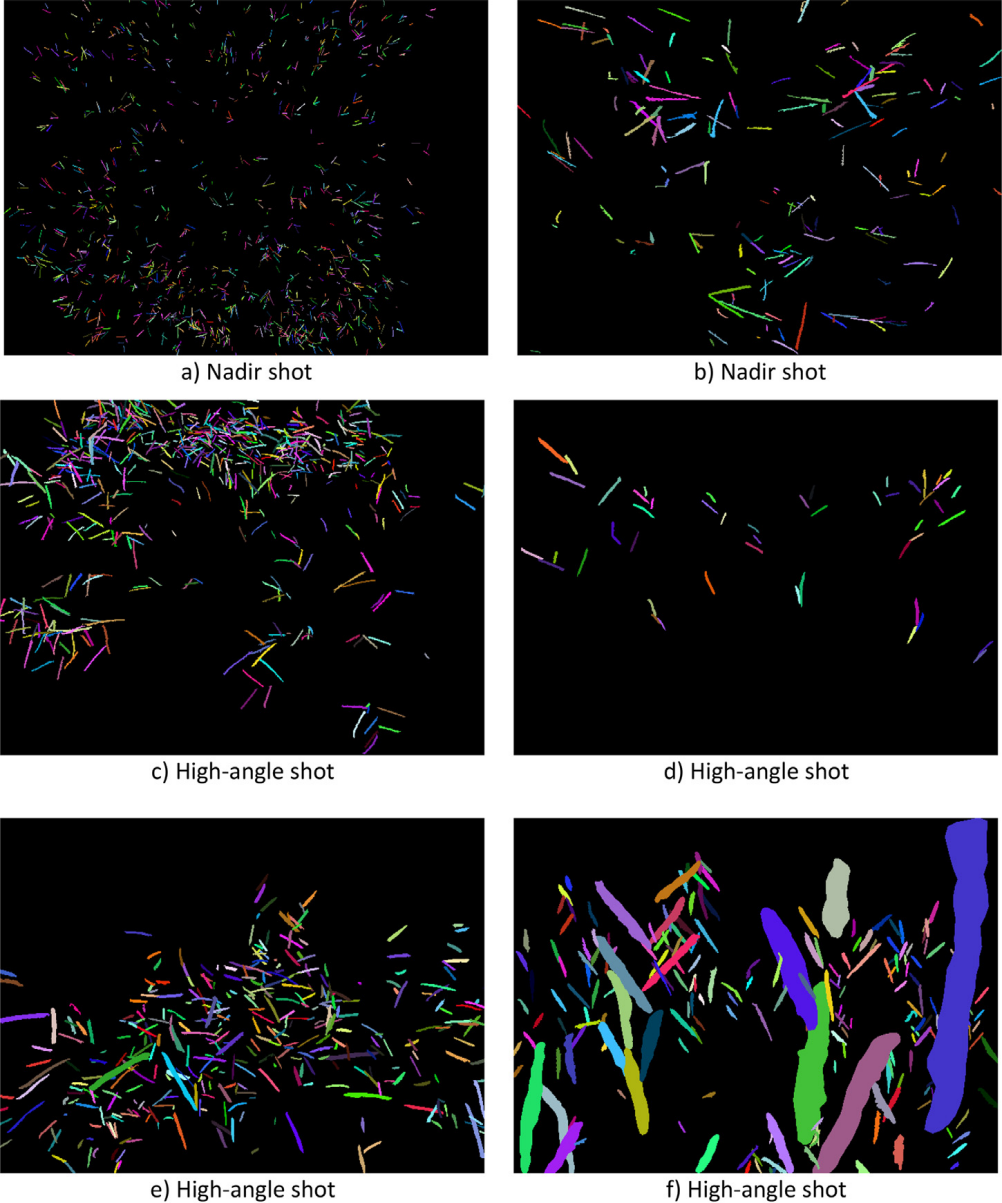
Corresponding Individual annotations of images in Fig. 2.

**Fig. 4:**
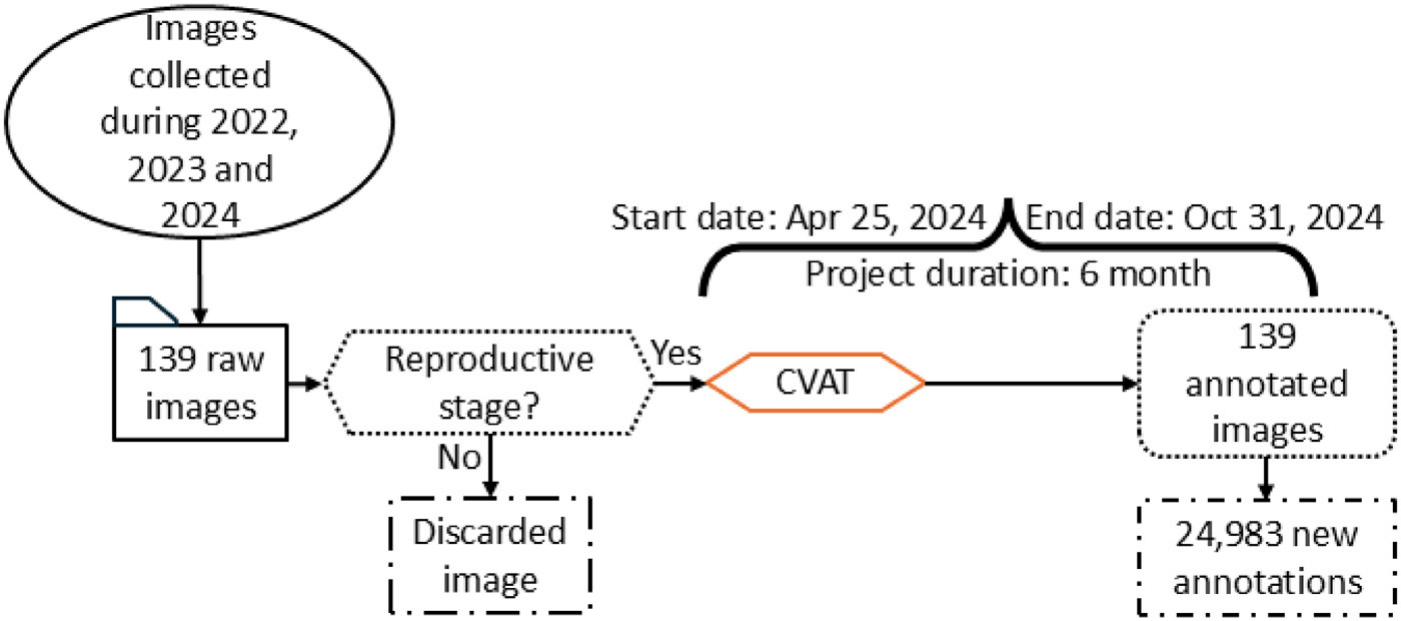
Data annotation process.

The expanded dataset is organized into three main files [2]. The first file, named “01.ReadMe_RGB_ImageDatasetOfUrochloaHybridsForHTP_and_AI_applications.txt”, is a plain text document that provides basic information about the dataset and usage guidelines. The second file is a folder called “02.Images.zip”, containing 139 new images. The third file, “03.RacemesInstances.json”, holds the 24,983 new annotations formatted according to the COCO (Common Objects in Context) standard, which is widely used for object detection and instance segmentation tasks. This format was chosen over others due to its flexibility, compatibility with most DL frameworks, and ability to store detailed information about object categories, bounding boxes, and segmentation masks. The images in this dataset have multiple pixel dimensions and capture settings, introducing variability in lighting, perspective, and spatial resolution. Table 1 details all the pixel dimensions and the corresponding number of images for each. There are 36 unique pixel dimensions in total, ranging from 488×645 to 5568×3712 pixels. This range reflects differences in image-capture devices and configurations, providing a diverse set of conditions. In contrast to the original dataset [1], some plants in this updated version were photographed in natural settings (see Fig. 2), including background vegetation like *Cynodon dactylon* [6] and *Paspalum notatum* [7], which can resemble racemes in appearance. This addition allows models trained on this dataset to better distinguish between racemes and similar-looking plants, an essential feature for practical field applications where background vegetation cannot always be removed. Fig. 3 shows the manual annotations of the images in Fig. 2, highlighting the high variability in the appearance of racemes. Although all racemes belong to *Urochloa* spp., they seem to display significant morphological differences due to the varied environmental conditions and image-capture configurations. The annotation process for all 139 new images was done using CVAT in the same way as the original dataset. However, the task became considerably more challenging because the density of the racemes increased. This led to a higher degree of occlusion and a longer annotation process. The original dataset took approximately four months to annotate. The updated dataset, despite having 116 fewer images to annotate, required about six months to complete (Fig. 4) and resulted in 2,643 more annotations than the original dataset (as shown in Fig. 1).

**Table 1:** Image specifications.

Dimension (width x height)	# Images	Dimension (width x height)	# Images
488×645	1	3785×2713	1
572×413	1	3881×2733	1
768×1024	2	4000×3000	6
2325×1517	1	4032×3024	2
2729×1613	1	4065×2737	1
3000×4000	2	4080×3072	27
3021×3135	1	4121×2713	1
3072×4080	3	4160×3120	2
3073×1961	1	4193×2321	1
3120×4160	5	4217×3249	1
3129×2145	1	4224×3168	16
3393×2081	1	4393×2257	1
3472×4624	1	4561×2801	1
3529×1977	1	4624×3472	6
3552×2664	5	4633×2529	1
3553×2489	1	5457×3681	1
3585×2641	1	5472×3648	4
3721×2577	1	5568×3712	36

## 4 Experimental Design, Materials and Methods

This dataset combines images from two distinct experimental locations: Palmira, Colombia, and Ocozocoautla de Espinosa, Mexico. The images from Palmira in both datasets (the initial release and this updated version) were captured at the same location: the International Center for Tropical Agriculture (CIAT) in Palmira, Colombia, as described in [3].

**Fig. 5:**
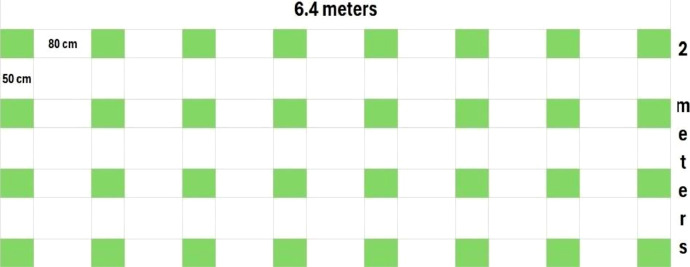
Experimental design of *Urochloa* hybrids plots where images were collected at CIPAT, Mexico. Green squares represent position and size of *Urochloa* plots.

The second set of images was collected as part of a phenotyping experiment conducted at the Tropical Forage Research Center (CIPAT) in Ocozocoautla de Espinosa, Chiapas, Mexico. This experiment followed a Randomized Complete Block Design (RCBD) with four replications. Each experimental unit consisted of 32 plants, spaced 80 cm x 50 cm, as shown in Fig. 5. The experimental units were subdivided into forage production and seed production sections, but photos were taken across the entire plot during the flowering stage to capture the full phenotypic expression of the genotypes. Multiple image capture devices were used, including a Nikon D5600 professional camera, Realme C53, Moto G7 Power, Galaxy A71 and Oppo Reno 11 smartphones, and a Phantom 4 Pro V2 drone. Each image capturing device has its own camera settings and sensor specifications. These factors influence key characteristics of the resulting images. Including: resolution, dynamic range, and color representation. All these devices introduce variability in the dataset related to sensor type and image quality. This kind of variability is useful when training DL models, as they benefit from exposure to images taken from different sources. The images were taken at distances ranging from 1 to 3 meters from the plant canopy to accommodate plants of varying sizes and to capture all the details of the racemes. The drone, equipped with a 1-inch 20 MP CMOS sensor, was flown at altitudes between 3 and 5 meters, using a mechanical shutter to prevent distortion and an f/2.8 wide-angle lens to capture accurate sharp and vivid images. Photos were taken outdoors at different times of the day, resulting in natural variations in lighting conditions, including changes in sunlight intensity and shadow patterns. This variability allows models to learn under conditions that are more reflective of field environments. The images were captured from multiples perspectives, including nadir, high-angle, and frontal views, enhancing the perceived morphological diversity of the plants. Although the actual morphology of the racemes remains the same, racemes may appear shorter, longer, wider, or narrower depending on the angle and distance from which the photos were taken, as shown in Fig. 2. This perceived variability is essential for creating DL models that can recognize phenotypic traits across different viewpoints and scales.

Raceme density per plant also increased. In the original dataset, the plant with the highest raceme count had 851 racemes. In this updated dataset, raceme counts reach as high as 1586 in a similar area (∼1m²), nearly doubling the count. This increase leads to a much higher degree of raceme overlapping.

## Limitations

Not applicable.

## Ethics Statement

Authors have read and follow the ethical requirements for publication in Data in Brief. The collected dataset does not involve human subjects, animal experiments, or any data collected from social media platforms.

## Credit Author Statement

**Darwin Alexis Arrechea-Castillo:** Conceptualization, data curation, formal analysis, writing— original draft, writing - review & editing; **Paula Espitia-Buitrago:** Experimental design, formal analysis, data curation, writing - review & editing; **David Florian-Vargas:** Experimental design, formal analysis, data curation, writing - review & editing; **Ronald David Arboleda**: Data curation, formal analysis; **Riquelmer Velázquez-Hernández:** Experimental design, data curation, formal analysis; **Andres Felipe Ruiz-Hurtado:** Formal analysis, writing - review & editing; **Luis Miguel Hernandez:** Review & editing; **Rosa N. Jauregui:** Review & editing, supervision, resources, funding acquisition; **Juan Andrés Cardoso:** Experimental design, data curation, formal analysis, writing—original draft, writing - review & editing, supervision, resources, funding acquisition.

## Data Availability

DataverseRGB Image Dataset of Urochloa Hybrids for High-Throughput Phenotyping and Artificial Intelligence Applications (Original data).
